# Supplementary material for: Primate lentiviruses require Inositol hexakisphosphate (IP6) or inositol pentakisphosphate (IP5) for the production of viral particles
Source: PLoS Pathog. 2020 Aug 10;16(8):e1008646. doi: 10.1371/journal.ppat.1008646 (PMC7446826; doi:10.1371/journal.ppat.1008646)
Supplement: Attachment — Submitted filename: Response to Reviewers Final mcj.docx [file ppat.1008646.s001.docx]

**Response to Reviewers** of “Inositol hexakisphosphate (IP6) and inositol pentakisphosphate (IP5) are required for the production of viral particles by primate lentiviruses”

We thank the reviewers for their thoughtful reviews and helpful suggestions.

**Part I – Summary (Major Points):**

1. It is hard to get from the paper what the major finding is. It is not fair to conclude that IP5 is required for particle production, as knock-out of the molecule has no effect in normal cells. An accurate statement of their findings might be that in the absence of IP6, IP5 can partially substitute. It is important that the authors make a clear statement as to the point of their study. This should be in the abstract and in the Discussion.

We have emphasized the conclusion that either IP6 or IP5 is an absolute requirement for viral production. See the rewritten Abstract (lines 27-28, 34-36), Author Summary (lines 54-59), last paragraph of the Introduction (lines 110-118), and Discussion (lines 333-336).

2. Mallery et al. recently showed that IP5 can substitute for IP6. Isn’t that at odds with the results here in which it is shown that IP5 only poorly substitutes for IP6? The authors need to clarify whether this is a discrepancy or can be explained.

See rewritten Introduction (lines 113-114), Results (226-231) and Discussion (lines 327-336, 380-389). Their results are not at odds with ours at all. They see a large reduction in viral release in the absence of IPPK, but the limited virus released was still infectious. This is very much in agreement with our findings.

3. One wonders whether the transient depletion of IP5/6 from the cells is having other effects that lead to the failure to assemble. These molecules play critical signaling roles in cells. Are the authors sure that the assembly deficiency is due to the absence of the molecules from the virion and not from another effect on the cell?

See rewritten Results (lines 218-226) and Discussion (lines 371-379). While there are cytopathic effects of IP5/6 depletion from cells (Fig 4C-D), we believe this only reduces infectious particle production by 2-3 fold. In support of this conclusion, other retroviruses (MLV and MPMV) seem largely unaffected by modulation of IP5/6 via IPPK-KO and transient expression of MINPP1 (Fig 7A-B).

4. Fig. 2F. IPPK knock-out prevents virus release. In these cells there should be a buildup of Gag Pr55. In fact, the cells show less Gag precursor. That would suggest that it’s not a block to assembly, but a block to Gag synthesis.

See rewritten Results (lines 160-166). Because our experiments are performed with a defective provirus that encodes Gag, conditions where infectious particles can be produced always leads to some enhancement in Gag expression due to reinfection of transfected cells. The experiment would need to be redesigned using a vector that does not contain the Gag gene to precisely determine if there is Gag buildup in IPPK-KO cells. However, this was not the primary objective of the experiment.

5. The earlier Mallery et al. Cell Reports paper shows that FIV requires IP6. That seems to be at odds with the results here. The authors need to comment on this apparent discrepancy.

See rewritten Discussion (lines 417-426). There is no discrepancy. We both see about a 3-fold reduction in FIV particle production. Our study went further by including transfections that included IPPK and MINPP1. Because neither of these additions further modulated FIV levels, we concluded that reduction was not IP5/6 related. The Mallery paper did not do IPPK addback experiments.

**Part II – Major Issues: Key Experiments Required for Acceptance**

*Reviewer #1:*

1. The tests for the effects on virion production are minimal here (really it’s all Fig 5C). We probably need error bars on this key experiment.

We generated a graph with data points of western data (5D-E).

2. Some of the presentation is a little confusing in its relationship to the earlier work. As the authors point out (line 106), “Knockouts of the IPPK and IPMK genes have both been reported to reduce the production of infectious HIV-1 particles in vivo.” Thus, work very similar to what we see here is in the literature (Cell Rep. 2019;29(12):3983 and Nature. 2018;560: 509). Still, there are some aspects of the present study that are new: We do get a deeper understanding of the relative importance of IP5 vs. IP6. And issues such as requirements for virus entry are addressed. Nonprimate viruses are examined. The paper acknowledges the earlier work but doesn’t make it totally clear what is old news and what is truly new information – we need a sharper transition.

See the rewritten Abstract (lines 25-28, 34-36), Author Summary (lines 54-59), last paragraph of the Introduction (lines 110-118), and Discussion (lines 325-336).

3. I think we need to be given more data on the nature of the KO mutations being studied here. 293 cells are notoriously aneuploid, and often have 3 or 4 or more copies of various chromosomes. Thus, the KO clones generated need to be characterized to know that all alleles were genuinely mutated, to know what the alleles are, and to be sure that no wild-type alleles or partially active alleles are still lurking. This all becomes more crucial when there is the potential for partial activity to explain the findings. On line 429 of methods – how many clones were tested to find good KOs, and how many clones were tested for virus (I gather only one)? How many alleles were examined in each cell clone? What were the alleles – any in-frame mutations? “Direct sequencing” of PCR products is not adequate, because it only gives the majority allele. There is a broad assumption here that CRISPR is perfect every time, and this is definitely not true.

See the rewritten Methods (lines 488-513) which contains much more details of how we screened for the cell lines. We screened >10 clonal isolates for each cell line. Amplifying the gRNA target site effectively screens all of the alleles. By directly sequencing the amplification one can identify all of the variable alleles, even if they are a very minor. For both IPPK and IPMK we selected clones in which all of the alleles contained a frame-shift. In addition, the chosen cell lines were verified phenotypically by analyzing the IP5/6 levels.

*Reviewer #2:* N/A

*Reviewer #3:* None

**Part III – Minor Issues: Editorial and Data Presentation Modifications**

*Reviewer #1:*

1. While the MINPP1 overexpression after transient transfection was apparently successful in eliminating detectable IP5 and IP6, giving a window to examine effects on virus, there is always the concern that the cells are potentially very sick – as indicated by the inability to recover stable transfectants. Given this situation, how confident can we be that the inhibition of virus production is not a problem with the general metabolic state of the cells, caused by the low IP5/6? One pretty good answer is the near normal virion release of MLV and MPMV. This point could be made more forcefully.

See rewritten Results (lines 218-226) and Discussion (lines 371-379). While there are cytopathic effects of IP5/6 depletion from cells (Fig 4C-D), we believe that the assembly deficiency with HIV-1 and SIV is due primarily to the absence of the IP5/6 from the virion. As the reviewer points out, the strongest argument of this is the fact that MLV and MPMV are largely unaffected by IP5/6 modulation. We have tried to state this more forcefully.

2. In the title, maybe viral particle “release” is the wrong word – it probably isn’t release per se that requires these compounds. Better would be viral particle “production” or something else. This is true elsewhere (l. 155 etc.).

We have changed the title to, "Inositol hexakisphosphate (IP6) and inositol pentakisphosphate (IP5) are required for the production of viral particles by primate lentiviruses."

3. In the abstract, line 31, one of the middle sentences would be clearer if it were changed to something like “…but transient expression of the enzyme multiple inositol polyphosphate phosphatase-1 (MINPP1) in IPPK-KOs….”

See rewritten Abstract (lines 32-34). Added “the enzyme” to the sentence.

4. Line 154: It’s probably too hard to measure low levels of CA, but it would be nice if the level of physical particles could be quantitatively compared with infectious virus, to confirm that the low levels of released virus are of normal infectivity. It’s certainly roughly true.

As the reviewer says, it becomes increasingly difficult to quantify very low CA levels. Mallery *et al.* has already published that the particles remain fully infectious and we are not challenging that finding.

5. Line 281: “neither virus” is unclear. The authors mean EIAV and FIV, but since in the last sentence MLV and MPMV are also mentioned, this needs to be reworded.

See rewritten Results (line 303). Changed beginning of sentence to “neither EIAV nor FIV viruses were…”

6. Some comments on the processing of Gag might be warranted. In the KO, Gag is apparently no longer processed. Would a protease minus Gag behave any differently in terms of virion yield in any of the experiments? Some speculation could be included.

See rewritten Discussion (lines 353-357). In short, we think if the virus never assembles, it never gets processed. Yes, if a protease defective GagPol were used we would probably see less GagPol amplification (because there wouldn't be any reinfection in the transfected cells) and we obviously wouldn't see an processing, but we would also lose the parallel infectivity data.

*Reviewer #2:*

1. As mentioned above, several key findings, e.g., the ability of IP5 to substitute for IP6 in virus assembly, and the lack of an infectivity defect in virus produced from IPPK KO cells (which unfortunately was not properly tested in the authors’ Nature paper because they measured particle infectivity, not particle assembly/release), were made in ref 31 but this seems to be a bit downplayed here.

We have referenced the Mallery manuscript numerous times to point out how our findings are in agreement with theirs, but that our studies have expanded on their findings.

2. The EIAV results would appear to contrast with what these authors recently published (Dick et al., PLoS Pathog 2020). This should be clarified for the reader.

See rewritten Discussion (lines 427-433). In short, though nuanced, the results are not conflicting. IP6 seems to stimulate EIAV, but is not a requirement.

*Reviewer #3:*

1. Fig. 2 B and C. Remove shading from histogram bars.

See figure. Removed shading.

2. Fig. 2 E. Error bars should be made more visible.

See figure. Made error bars more prominent.

3. Fig. 4B. typo on “transduce” should be “transduced”.

See figure. Changed tenses.

4. Fig. 5B. the log scale on the Y axis showing the tick marks looks weird. Also in other figures. Remove this.

See figures. Removed center tick marks.
